# Filarial Lymphedema Patients Are Characterized by Exhausted CD4^+^ T Cells

**DOI:** 10.3389/fcimb.2021.767306

**Published:** 2022-01-06

**Authors:** Sacha Horn, Manuel Ritter, Kathrin Arndts, Dennis Borrero-Wolff, Anna Wiszniewsky, Linda Batsa Debrah, Alexander Y. Debrah, Jubin Osei-Mensah, Mkunde Chachage, Achim Hoerauf, Inge Kroidl, Laura E. Layland

**Affiliations:** ^1^ Division of Infectious Diseases and Tropical Medicine, University Hospital Munich, Ludwig-Maximilians-Universität (LMU), Munich, Germany; ^2^ Institute for Medical Microbiology, Immunology and Parasitology (IMMIP), University Hospital Bonn (UKB), Bonn, Germany; ^3^ Filariasis Unit, Kumasi Centre for Collaborative Research in Tropical Medicine (KCCR), Kumasi, Ghana; ^4^ Department of Clinical Microbiology, School of Medicine and Dentistry, Kwame Nkrumah University of Sciences and Technology, Kumasi, Ghana; ^5^ German-West African Centre for Global Health and Pandemic Prevention (G-WAC), Partner Site, Kumasi, Kumasi, Ghana; ^6^ Faculty of Allied Health Sciences, Kwame Nkrumah University of Sciences and Technology, Kumasi, Ghana; ^7^ Department of Immunology, National Institute for Medical Research (NIMR)-Mbeya Medical Research Center (MMRC), Mbeya, Tanzania; ^8^ Department of Microbiology and Immunology, University of Dar es Salaam-Mbeya College of Health and Allied Sciences (UDSM-MCHAS), Mbeya, Tanzania; ^9^ German-West African Centre for Global Health and Pandemic Prevention (G-WAC), Partner Site, Bonn, Bonn, Germany; ^10^ German Centre for Infection Research (DZIF), Neglected Tropical Disease, Partner Site, Bonn-Cologne, Bonn, Germany; ^11^ German Centre for Infection Research (DZIF), Neglected Tropical Disease, Partner Site, Munich, Munich, Germany

**Keywords:** filariae, CD4^+^ T cell exhaustion, *Wuchereria bancrofti* infection, lymphatic filariasis, immune modulation, lymphedema

## Abstract

Worldwide, more than 200 million people are infected with filariae which can cause severe symptoms leading to reduced quality of life and contribute to disability-adjusted life years (DALYs). In particular, lymphatic filariasis (LF) caused by *Wuchereria bancrofti* can lead to lymphedema (LE) and consequently presents a serious health problem. To understand why only a fraction of the infected individuals develop pathology, it is essential to understand how filariae regulate host immunity. The central role of T cells for immunity against filariae has been shown in several studies. However, there is little knowledge about T cell exhaustion, which causes T cell dysfunction and impaired immune responses, in this group of individuals. Recently, we showed that LE patients from Ghana harbor distinct patterns of exhausted effector and memory CD8^+^ T cell subsets. Based on these findings, we now characterized CD4^+^ T cell subsets from the same Ghanaian patient cohort by analyzing distinct markers within a 13-colour flow cytometry panel. We revealed that LE patients had increased frequencies of CD4^+^ T cells expressing exhaustion-associated receptors such as KLRG-1, TIM-3 and PD-1 compared to healthy endemic normal and *W. bancrofti*-infected individuals. Moreover, CD4^+^ T cells in LE patients were characterized by distinct co-expression patterns of inhibitory receptors. Collectively with the previous findings on CD8^+^ T cell exhaustion patterns, the data shown here demonstrates that filarial LE patients harbor distinct subsets of exhausted T cells. Thus, T cell exhaustion patterns in LE patients need attention especially in regards to susceptibility of concomitant infections and should be taken into consideration for LE management measures.

## Introduction

The Global Programme to Eliminate Lymphatic Filariasis (GPELF), implemented more than 20 years ago, includes mass drug administration (MDA) and vector control programmes. Although it has reduced the prevalence of infection in endemic areas, lymphatic filariasis (LF) remains endemic in several low-middle-income countries, particularly in Africa (Ramaiah and Ottesen, 2014; World Health Organization, 2020; World Health Organisation 2021). *Wuchereria bancrofti*, the causative agent of LF in Africa, has been shown to modulate the human immune system by promoting Th2 immune responses, alternatively activated macrophages and regulatory T and B cell subsets (Babu et al., 2006; Babu et al., 2009; Metenou et al., 2010; Ritter et al., 2019; Wammes et al., 2012). Due to filarial-driven immune modulation, the majority of infected individuals remain asymptomatic, presenting unique immune-profiles but appears to have a reduced immunity against viral, bacterial or other parasitic infections (Arndts et al., 2012; Chatterjee et al., 2014; George et al., 2014; Kroidl et al., 2016; Kwan et al., 2018). Despite the tightly controlled immune-regulation, individuals with LF can also develop severe symptoms like hydrocele or lymphedema (LE) as well as adenolymphangitis (ADL) attacks (World Health Organization, 2020; World Health Organisation 2021). This often leads to reduced quality of life and increased disability-adjusted life years (DALYs), financial losses, and social separation due to the stigmatization (Gyapong et al., 1996; van 't Noordende et al., 2020; Asiedu et al., 2021). Interestingly, the majority of patients suffering from severe pathology have usually cleared the infection and their profiles are characterized by increased antigen-specific Th1 and Th17 responses and constant immune activation (Babu et al., 2009; Babu and Nutman, 2012; Babu and Nutman, 2014). Recently, we showed that CD4^+^ and CD8^+^ T cells are activated during *W. bancrofti* infection (Kroidl et al., 2019). It is known that constant immune activation accompanied with persistent antigen loads and inflammation can lead to T cell exhaustion, which is defined as T cell dysfunction accompanied with impaired effector function and expression of distinct inhibitory receptors (Wherry, 2011; Wherry and Kurachi, 2015). Important receptors and markers that are associated with such exhaustion include programmed cell death-1 (PD-1), lymphocyte activation gene 3 (LAG-3), killer cell lectin-like receptor subfamily G member 1 (KLRG-1), cluster of differentiation 39 (CD39) and T-cell immunoglobulin and mucin-domain containing-3 (TIM-3) (Wherry, 2011; Gupta et al., 2015; Wherry and Kurachi, 2015; Dong et al., 2019). By using advanced flow cytometry to explore the expression of these exhaustion-associated markers on T cells, we recently showed that patients with LE due to LF from Ghana harbour distinct exhaustion patterns of memory and effector CD8^+^ T cell subsets compared to *W. bancrofti*-infected individuals and healthy endemic controls (Horn et al., 2021). Expanding on these findings, we further investigated the exhaustion patterns on CD4^+^ T cell subsets from *W. bancrofti*-infected individuals, healthy controls and individuals that suffer from LE and revealed an increased expression of these markers on CD4^+^ T cells from patients presenting clinical pathology.

## Methods

### Ethics

Participants were recruited from the Upper East Region of Ghana (Navrongo, Kassena-Nankana Municipal District) in 2018 as part of the ongoing German Federal Ministry of Education and Research (BMBF) funded TAKeOFF LEDoxy clinical trial or the German Research Foundation (DFG) funded RHINO project. All participants were over 18 years of age at the time of recruitment and all gave written informed consent before blood collection. Ethical approval was obtained from the Ethics Committee of the LMU Munich, Germany (17-858, LEDoxy) and (18-377, RHINO), the Ethics Committee at the University Hospital of Bonn, Germany (359/17, LEDoxy) and (041/18, RHINO), the Committee on Human Research Publication and Ethics at the Kwame Nkrumah University of Science and Technology in Kumasi, Ghana (CHRPE/AP/525/17, LEDoxy, CHRPE/AP/235/18, RHINO), the Ghana Food and Drugs Authority (FDA/CT/181 and FDA/CT/181(1)) and the Ghana Health Services (GHS-ERC-007/07/17).

### Study Population and Parasitic Assessment

Blood samples were collected from uninfected healthy individuals considered to be endemic normals (EN), *Wuchereria bancrofti*-infected individuals (Wb-inf.), and individuals with filarial LE on the legs for advanced flow cytometric analysis. At the time of sample collection, an epidemiologically-based survey was given to participants including questions about gender, age, the number of years they had lived in filarial-endemic areas, and the number of times medication (ivermectin and albendazole) was taken as part of MDA programmes. All study participants were tested with the Filariasis Test Strip (FTS; previously Alere, now Abbott Laboratories, Chicago, USA) and the TropBio Og4C3 Filariasis Antigen ELISA (hereafter referred to as TropBio, Cellabs, Brookvale, Australia). Participants in the EN cohort were negative for both FTS and TropBio tests, had been living in the filarial-endemic area for a minimum of 5 years and had no visible signs of LE. The Wb-infected cohort was positive for both FTS and TropBio tests and had no signs of LE. The LE cohort tested negative for both antigen tests and the extent of the lymphedema was classified according to the Dreyer staging system (Dreyer et al., 2002). Despite presenting lymphedema, all participants were considered to be in general good health with no clinical signs of other infections.

### Advanced Flow Cytometry to Characterize CD4^+^ T Cell Exhaustion

As part of an ongoing field study in northern Ghana, peripheral blood mononuclear cells (PBMC) were isolated and cryopreserved in liquid nitrogen, as previously described (Arndts et al., 2012; Arndts et al., 2015; Horn et al., 2021). The cells were then transported to Germany where they were thawed and washed twice with RPMI 1640 medium supplemented with 10% FCS, gentamycin, penicillin/streptomycin (all 50 µg/mL) and L-glutamine (292.3 µg/mL) (Sigma-Aldrich, St. Louis, USA). Next, cells were permeabilized with the FoxP3 Fixation/Permeabilization kit, as per the manufacturer’s instructions (Thermo Fisher Scientific, Life Technologies Corporation, Grand Island, USA). After permeabilization, cells were incubated at 4°C for 20 minutes with a 13-colour anti-human antibody panel including CD4-BUV661 (clone SK3), CD8-BUV395 (clone HIT8a), CD39-BV 510 (clone TU66) (all obtained from BD™ Biosciences), IFN-γ-FITC (clone 4S.B3), IL-10-PE (JES3-9D7), T-bet-PE-Cy7 (clone 4B10), Eomes-PE-eFluor 610 (clone WD1928), PD-1-APC-eFluor 780 (clone eBoJ105), LAG-3-eFluor 450 (clone 3DS223H), TIM-3-Super Bright 600 (clone F38-2E2), KLRG-1-PerCP-eFluor 710 (clone 13F12F2), CD127-AF700 (clone eBioRDR5), and TNF-α-APC (clone Mab11). Cells were then washed twice with the permeabilization buffer before finally being resuspended in 100 µL of PBS. All data were acquired using the CytoFlex S flow cytometer (Beckman Coulter, Brea, USA) and analysis was performed with FlowJo_v10.6.0 software (FlowJo LLC, Ashland, Oregon, USA). The gating strategy was developed using fluorescence minus one controls and the compensation was done with the VersaComp Antibody Capture Kit (Beckman Coulter). Only samples containing greater than 2,000 CD4^+^ T cell events were included to avoid analysis of artefacts. Boolean gating was applied in order to analyse the co-expression of multiple inhibitory receptors on CD4^+^ T cell subsets. All of the supplements, reagents, and media used were from Thermo Fisher Scientific, unless otherwise specified.

### Statistical Analysis

Statistical analysis was performed using SPSS software (IBM SPSS Statistics 22, Armonk, NY), CRAN R 3.6.2, and the GraphPad Prism 6.01 programme (GraphPad Software, Inc., La Jolla, USA). According to the Kolmogorov-Smirnov test, all variables showed non-parametric distribution and therefore the Kruskal-Wallis test for multiple comparison followed by Dunn’s *post hoc* test was applied for further comparison between the groups. P-values were considered statistically significant below 0.05. For comparisons of continuous parameters, the Spearman correlation was used.

## Results

### Filarial Lymphedema Patients Harbour Exhausted CD4^+^ T Cell Subsets

Due to our recent findings that Ghanaian individuals presenting LF-induced LE are characterized by distinct patterns of exhausted effector and memory CD8^+^ T cell subsets (Horn et al., 2021), we investigated whether CD4^+^ T cell subsets were also affected. In short, we investigated exhaustion patterns on CD4^+^ T cell subsets from the same Ghanaian cohort (EN, Wb-inf. and LE) by analyzing the distinct markers CD4, T-bet, Eomes, CD127, PD-1, KLRG-1, TIM-3, LAG-3, CD39, TNF-α, IL-10 and IFN-γ from a 13-colour flow cytometry panel. An overview about the characteristics of the study population is shown in **Table 1**.

**Table 1:** Study population characteristics.

Characteristics	EN	Wb-inf.	LE
Sample size (n)	44	31	26
Median age [range]	43.17 [20-75]	43.32 [20-75]	46.58 [26-65]
Gender (female:male) [%]	29:15 [66:34]	16:15 [52:48]	22:4 [85:15]
Mean years living in the endemic area [range]	40.10 [6-75]	39.96 [6-75]	46.58 [26-65]
Median MDA rounds [range]	4 [0-15]	4 [0-15]	5 [2-15]
Median lymphedema stage [SD]	NA	NA	3 [1.70]
FTS result/TropBio result	-/-	+/+	-/-

Study participants were characterized as endemic normals (EN), considered to be healthy individuals, Wuchereria bancrofti-infected (Wb-inf.) or patients with lymphedema pathology (LE). EN participants were negative for both the Filariasis Test Strip (FTS) and TropBio Og4C3 Filariasis Antigen ELISA (TropBio), Wb-infected participants were positive for both the FTS and TropBio tests, and the LE group was defined by the presence of pathology which was classified according to the Dreyer staging protocol (Dreyer et al., 2002). **Table 1** summarizes total sample size, age, gender, years living in the endemic area, as well as mean rounds of MDA received and median lymphedema stage. NA, not applicable.

**Figure 1:**
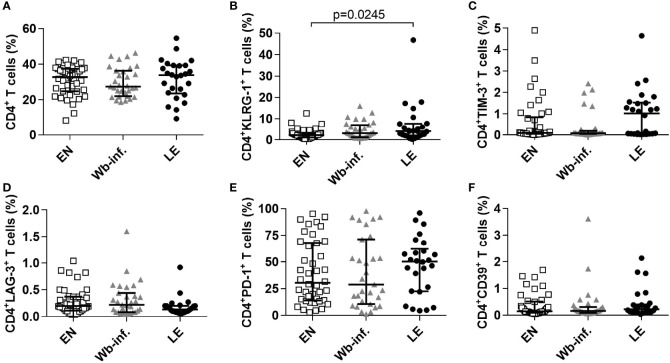
Elevated frequencies of exhausted CD4^+^ T cell subsets in filarial LE patients. Frequencies of CD4^+^ T cells were measured by flow cytometry in PBMCs from cohorts of healthy endemic normal subjects (EN, n=44), *Wuchereria bancrofti*-infected (Wb-inf., n=31) and lymphedema patients (LE, n=26). **(A)** Cell populations were analyzed according to the applied gating strategy (**Supplementary Figure S1**) to decipher frequencies of **(A)** CD4^+^, **(B)** CD4^+^KLRG1^+^, **(C)** CD4^+^TIM-3^+^, **(D)** CD4^+^LAG-3^+^, **(E)** CD4^+^PD-1^+^ and **(F)** CD4^+^CD39^+^ T cell subsets. Symbols in graphs show individual data sets with median and interquartile range. Statistical significance between the groups was obtained using Kruskal-Wallis followed by Dunn’s multiple comparison *post hoc* analysis.

To determine levels of exhausted effector (T-bet^hi^Eomes^dim^) and memory (T-bet^dim^Eomes^hi^) CD4^+^ T cells, we used our previously described gating strategy (Horn et al., 2021), but observed no differences in the frequencies of these cell subsets as well as CD127 expression or their cytokine levels (TNF-α, IL-10 and IFN-γ) between the three patient cohorts (data not shown). However, after applying the gating strategy which excluded the transcription factors Tbet and Eomes as depicted in **Supplementary Figure S1**, CD4^+^ T cells did show distinct exhaustion patterns in the LE cohort. In detail, whereas frequencies of CD4^+^ T cells were comparable between EN, *W. bancrofti*-infected and LE individuals (**Figure 1A**), frequencies of CD4^+^ T cells that express the exhaustion-associated receptor KLRG-1 were significantly increased in LE patients when compared to EN (**Figure 1B**). Moreover, CD4^+^ T cells from LE patients showed a tendency to increased frequencies in TIM-3 and PD-1 expression, whereas LAG-3 and CD39 remain comparable between EN and *W. bancrofti*-infected individuals (**Figures 1C–F**). Since the sample size in each stage (stages 2, 3, and 6) was too low for proper statistical analysis, we performed correlation analysis with pathology *per se*. Interestingly, these correlations reflected the results shown in **Figure 1**, since whereas frequencies of CD4^+^CD39^+^ (r=0.1230 and p=0.2228), CD4^+^LAG-3^+^ (r=0.0252 and p=0.8033) and CD4^+^PD-1^+^ T cells (r=0.1348 and p=0.1813) did not show significant correlation, frequencies of CD4^+^KLRG-1^+^ (r=0.2430 and p=0.0149) and CD4^+^TIM-3^+^ (r=0.2216 and p=0.0267) T cell subsets were positively correlated to LE pathology. Overall, these findings show that patients suffering from leg pathology are characterized by both exhausted CD4^+^ (**Figure 1**) and CD8^+^ T cells (Horn et al., 2021).

### Distinct Co-Expression Patterns of Exhaustion Receptors in Filarial Lymphedema Patients

**Figure 2:**
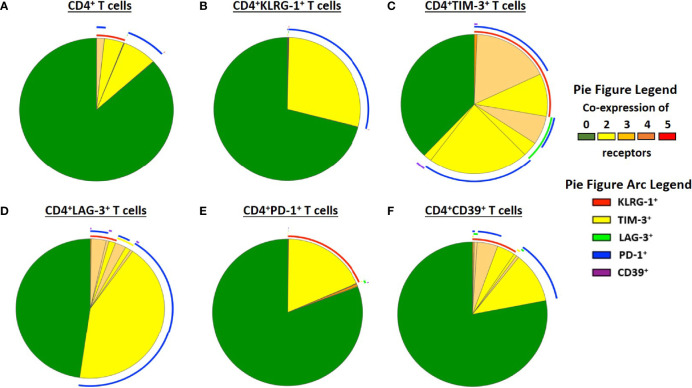
Co-expression patterns of receptors that are associated with exhaustion in filarial LE patients. Co-expression of exhaustion associated receptors in the lymphedema cohort (LE, n=26) was analysed on **(A)** CD4^+^, **(B)** CD4^+^KLRG1^+^, **(C)** CD4^+^TIM-3^+^, **(D)** CD4^+^LAG-3^+^, **(E)** CD4^+^PD-1^+^ and **(F)** CD4^+^CD39^+^ T cell subsets using Boolean gating.

Since exhaustion of T cells is characterized by co-expression of multiple inhibitory receptors (Wherry, 2011; Wherry and Kurachi, 2015), we further analysed co-expression of the exhaustion-associated receptors in the patient cohorts using Boolean gating. In general, receptor expression was comparable between the patient cohorts with slightly increased co-expression patterns of CD4^+^ TIM-3^+^ T cells in EN and CD4^+^LAG-3^+^, PD-1^+^ and CD39^+^ T cell subsets in the LE cohort (**Supplementary Figure S2**). However, detailed analysis of the co-expression of the exhaustion associated receptors in the LE cohort revealed that T cell subsets mainly co-express PD-1 and KLRG-1 (**Figure 2**). In addition, CD4^+^TIM-3^+^ T cells and CD4^+^LAG-3^+^ T cells co-expressed a variety of exhausted-associated receptors, namely PD-1, KLRG-1, LAG-3 or TIM-3 and in small proportions CD39 (**Figures 2C, D**). In addition, CD4^+^CD39^+^ T cells are characterized by co-expression of KLRG-1, PD-1 and partially TIM-3 and LAG-3 (**Figure 2F**). In summary, these findings show that LE patients have increased frequencies of exhausted CD4^+^ T cell subsets that co-express different inhibitory receptors, suggesting that patients with clinical pathology due to LF might have impaired T cell effector function which needs attention especially in regards to susceptibility of concomitant infections and LE management measures.

## Discussion

*Wuchereria bancrofti*-driven immunomodulation is a key feature of this helminth’s survival and homeostasis of host’s immune responses to maintain an asymptomatic disease state (Hoerauf et al., 2005; Babu et al., 2006; Babu et al., 2009; Adjobimey and Hoerauf, 2010; Babu and Nutman, 2012; Wammes et al., 2012; Rajamanickam and Babu, 2013; Metenou et al., 2010; Ritter et al., 2019). However, severe clinical outcomes such as LE can lead to increased DALYs and social stigmatisation (Gyapong et al., 1996; van 't Noordende et al., 2020; World Health Organization, 2021; World Health Organization, 2020; Asiedu et al., 2021). Several studies already characterized CD4^+^ T cells in LE patients and revealed that frequencies of naive, effector memory and central memory CD4^+^ T cells in patients with filarial disease and chronic infections were not different. However, LE patients are characterized by enhanced Th1 and Th17 immune responses accompanied with decreased Th2 cells and increased pro-inflammatory cytokines, chemokines and growth factors that led in the majority of the LE individuals to a clearance of the infection (Debrah et al., 2006; Satapathy et al., 2006; Babu et al., 2009; Bennuru and Nutman, 2009; Bennuru et al., 2010; Babu and Nutman, 2012; Babu and Nutman, 2014). Since filarial infections are chronic diseases, the immune system is constantly encountering filarial-derived antigens which have been recently shown to systemically activate CD4^+^ and CD8^+^ T cell subsets (Kroidl et al., 2019). Such constant activation can lead to T cell-intrinsic regulation, hypo-responsiveness and exhaustion which is important for immune tolerance and control of immune responses (Schwartz, 2003; Wherry, 2011; Wherry and Kurachi, 2015). This has also been observed during parasitic infections with intestinal helminths (Borkow et al., 2000; Leng et al., 2006), murine schistosomiasis (Taylor et al., 2009), *Fasciola hepatica* (Aldridge and O’Neil, 2016; Sachdev et al., 2017) and *Echinococcus multilocularis* (Wang et al., 2017; Zhang et al., 2017). Moreover, T cell hypo-responsiveness has been observed during filarial infections with the murine filaria *Litomosoides sigmodontis* including a crucial role of PD-1 (Taylor et al., 2005; van der Werf et al., 2013; Campbell et al., 2018; Knipper et al., 2019), which is, as mentioned earlier, an important receptor associated with T cell exhaustion when accompanied by LAG-3, KLRG-1, TIM-3 and CD39 (Wherry, 2011; Gupta et al., 2015; Wherry and Kurachi, 2015; Dong et al., 2019). Recently, we revealed that filarial LE patients harbour distinct exhausted effector and memory CD8^+^ T cells that were characterized by the expression of TIM-3, LAG-3, CD39, PD-1 and KLRG-1 (Horn et al., 2021). Expanding on those data, we show here that CD4^+^ T cell subsets from filarial LE individuals also have increased co-expression of exhausted associated receptors. However, further characterization of the CD4^+^ T cell subsets like central memory, effector memory and naïve T cells needs to be performed in future studies to decipher the mechanisms of exhaustion and T cell-specific cytokine responses in patients with disease sequelae. Indeed, an important limitation of the study was that not enough peripheral whole blood could be obtained from the patient cohorts to isolate sufficient amounts of PMBCs to apply another flow cytometry panel and perform re-stimulation experiments for further discrimination of T cell subsets and their cytokine responses, respectively. Nevertheless, the results that were obtained highlight the fact that T cells from individuals who suffer from lymphedema are exhausted and might then in turn lead to impaired immune responses to concomitant infections. Indeed, studies have shown that lymphedema patients have a higher risk for bacterial infection (Dupuy et al., 1999; Dean et al., 2020). It is also known that filarial LE individuals suffer from secondary infections which can cause acute dermatolymphangioadenitis attacks (ADLA) that drive progression of lymphedema (Schacher and Sahyoun, 1967; Olszewski et al., 1993; Shenoy et al., 1995; Olszewski et al., 1997). In summary, the T cell exhaustion patterns described here might be another factor as to why LE patients have a higher risk for bacterial infections and consequently suffering from ADLA. These findings need to be taken into consideration for prevention and control management of filarial lymphedema, with a particular focus on hygiene control management of the affected limbs.

## Data Availability Statement

The original contributions presented in the study are included in the article/**Supplementary Material**. Further inquiries can be directed to the corresponding author.

## Ethics Statement

The studies involving human participants were reviewed and approved by Ethics Committee of the LMU Munich, Germany (17-858, LEDoxy) and (18-377, RHINO), the Ethics Committee at the University Hospital of Bonn, Germany (359/17, LEDoxy) and (041/18, RHINO), the Committee on Human Research Publication and Ethics at the Kwame Nkrumah University of Science and Technology in Kumasi, Ghana (CHRPE/AP/525/17, LEDoxy, CHRPE/AP/235/18, RHINO), the Ghana Food and Drugs Authority (FDA/CT/181 and FDA/CT/181(1)) and the Ghana Health Services (GHS-ERC-007/07/17). The patients/participants provided their written informed consent to participate in this study.

## Author Contributions

LB, AYD, MC, IK, and LEL conceived and designed the study. SH, DB-W, AW, IK, LB, AYD, JO-M, and KA organized field studies and acquired samples. SH, DB-W, AW, MR, KA, and LEL processed samples, performed analysis, and interpreted the data. SH, IK and MR drafted the manuscript while LB, AYD, JO-M, AW, MC, and AH critically revised the article and controlled the intellectual content. All authors contributed to the article and approved the submitted version.

## Funding

This work was supported by the German Research Foundation (DFG) within the German-African Projects in Infectiology(RHINO project) [KR3615/1-1 and HO2009/11-1] to AH and IK. Furthermore, AH and IK are also supported by the German Federal Ministry of Education and Research (BMBF) [01KA1601] and as members of the German Centre of Infectious Disease (DZIF) [TI.03.907]. AH is additionally funded by the Deutsche Forschungsgemeinschaft (DFG, German Research Foundation) under Germany’s Excellence Strategy – EXC2151 – 390873048. Finally, MR is financially supported by the BMBF and the Ministry of Culture and Science of the State of North Rhine-Westphalia (MKW) within the framework of the Excellence Strategy of the Federal and State Governments.

## Conflict of Interest

The authors declare that the research was conducted in the absence of any commercial or financial relationships that could be construed as a potential conflict of interest.

## Publisher’s Note

All claims expressed in this article are solely those of the authors and do not necessarily represent those of their affiliated organizations, or those of the publisher, the editors and the reviewers. Any product that may be evaluated in this article, or claim that may be made by its manufacturer, is not guaranteed or endorsed by the publisher.
