# Supplementary material for: Filarial Lymphedema Patients Are Characterized by Exhausted CD4+ T Cells
Source: Front Cell Infect Microbiol. 2022 Jan 6;11:767306. doi: 10.3389/fcimb.2021.767306 (PMC8770542; doi:10.3389/fcimb.2021.767306)
Supplement: Supplementary file 1 [file DataSheet_1.docx]

Supplementary Material


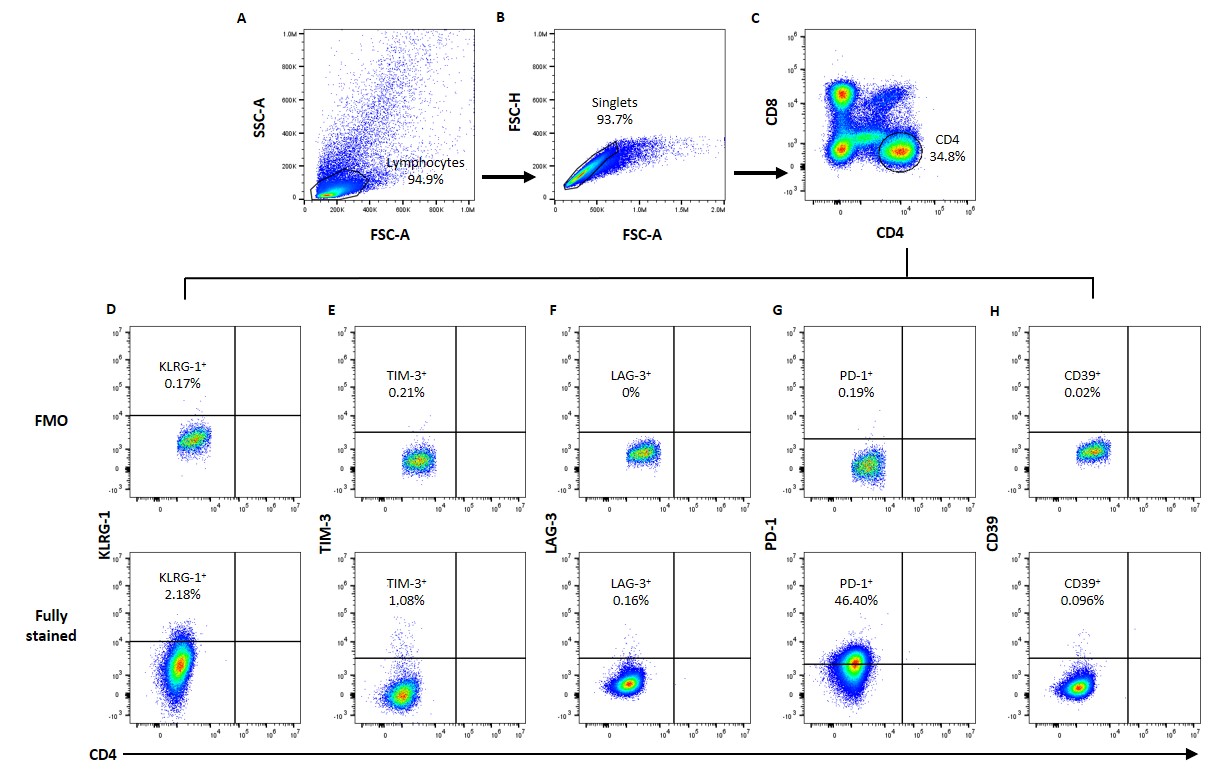


**Supplementary Figure S1: Gating strategy for exhausted associated receptors on CD4^+^ T cells**

**(A)** PBMCs were first gated to discriminate lymphocytes using forward-scatter (FSC) versus side scatter (SSC). Then, **(B)** single cells and **(C)** CD4 expression was analysed. Finally, expression of the inhibitory receptors **(D)** KLRG-1, **(E)** TIM-3, **(F)** LAG-3, **(G)** PD-1 and **(H)** CD39 on CD4^+^ T cells were analysed to discriminate the frequencies of exhausted CD4^+^ T cells subsets based on FMO controls.


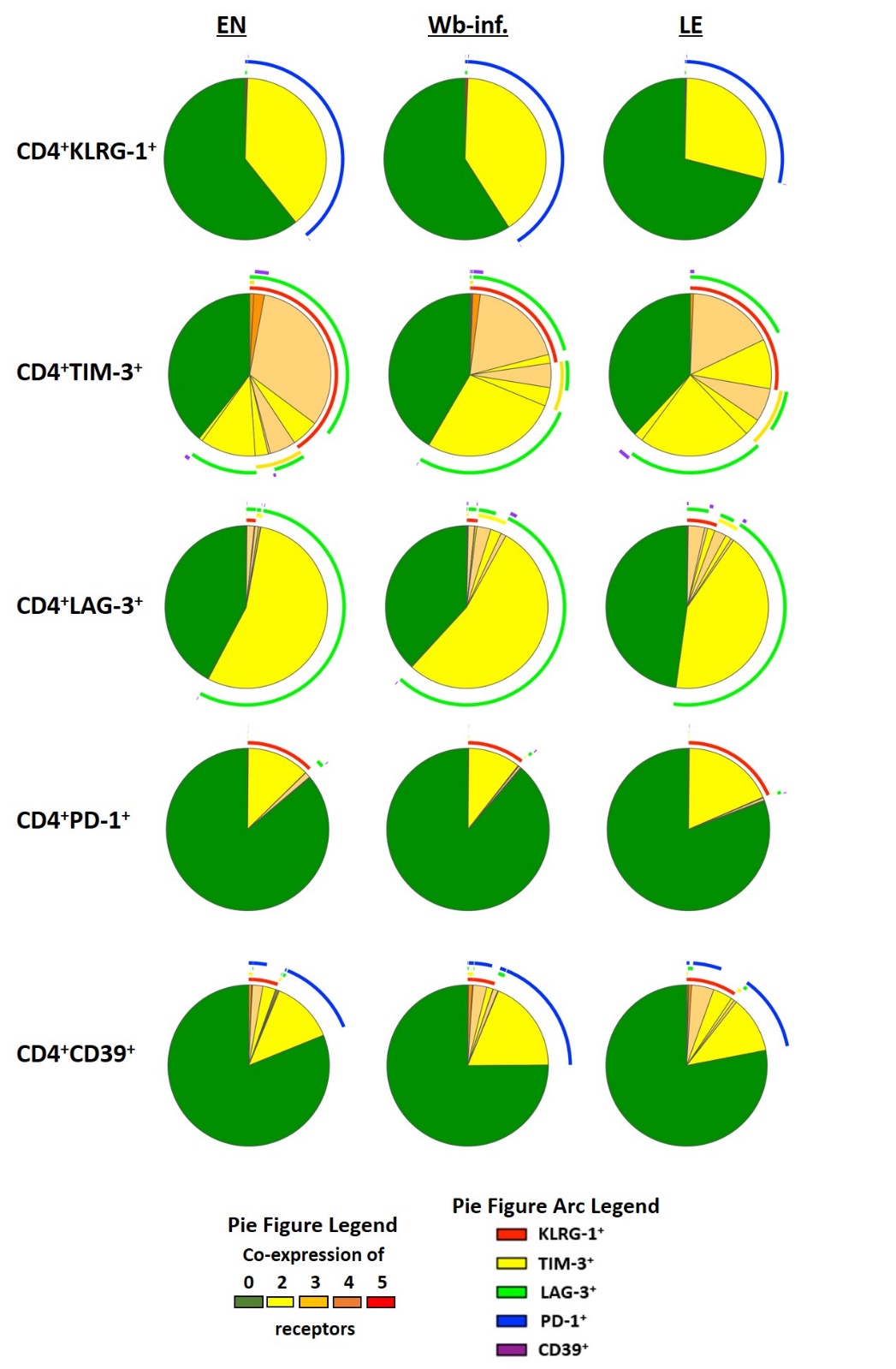


**Supplementary Figure S2: Co-expression patterns of exhausted associated receptors in the Ghanaian patient cohorts**

Co-expression of exhaustion associated receptors in healthy endemic normal subjects (EN, n=44), *Wuchereria bancrofti*-infected (Wb-inf., n=31) and lymphedema patients (LE, n=26) was analysed on CD4^+^KLRG1^+^, CD4^+^TIM-3^+^, CD4^+^LAG-3^+^, CD4^+^PD-1^+^ and CD4^+^CD39^+^ T cell subsets using Boolean gating.
